# The Interactional Characterization of Lentil Protein Isolate (LPI) with Cyanidin-3-O-Glucoside (C3G) and Their Effect on the Stability and Antioxidant Activity of C3G

**DOI:** 10.3390/foods12010104

**Published:** 2022-12-25

**Authors:** Hongxia Qian, Fanghua Guo, Hua Xiong, Hua Zhang, Li Jiang, Yong Sun

**Affiliations:** 1 State Key Laboratory of Food Science and Technology, Nanchang University, Nanchang 330047, China; 2 School of Pharnacy, Jiangxi University of Traditional Chinese Medicine, Nanchang 330004, China

**Keywords:** fluorescence spectroscopy, static quenching, hydrophobic effects, thermal and oxidation stability

## Abstract

The interaction between lentil protein isolate (LPI) and cyanidin-3-O-glucoside (C3G) was investigated via with UV–vis spectroscopy, circular dichroism, and fluorescence spectroscopy and the stability of anthocyanin was also evaluated. After LPI mixed with C3G, the turbidity and foaming capacity increased and the particle size and surface charge did not change significantly, while the surface hydrophobicity decreased significantly (*p* < 0.05). The fluorescence results indicated that C3G quenched the intrinsic of LPI by static quenching and LPI bound with C3G via hydrophobic effects with Ka of 3.24 × 106 M^−1^ at 298 K. The addition of LPI significantly (*p* < 0.05) slightly decreased the thermal and oxidation degradation of C3G by up to 90.23% and 54.20%, respectively, while their antioxidant activity was inhibited upon mixing. These alterations of physicochemical properties might be attributed to their structural changes during the interaction. The obtained results would be of help in stabilizing bioactive compounds and the development of functional foods.

## 1. Introduction

Anthocyanins, existing in vegetables, fruits, flowers, and leaves, have been extensively studied as an important natural active ingredient (polyphenols) [1]. Of the 635 anthocyanins known, cyanidin, delphinidin, peonidin, petunidin, malvidin, pelargonidin, and their glycosides are the most common [2]. Anthocyanins possess neuroprotective and anti-cardiovascular disease activities, eye and brain health properties, and anti-diabetic activities [3]. Among them, cyanidin-3-glucoside (C3G) showed promising anti-oxidation [4], anti-inflammation [5], and anti-obesity [6] potential. Therefore, anthocyanins, especially C3G, are already used as the functional ingredients or colorants/antioxidants in snacks and dairy products [7]. However, anthocyanins are subjected to chemical degradation under the influence of oxygen, temperature, light, pH, enzymes, carbohydrates, proteins, and minerals during food processing and storage, which might contribute to a loss of original colour and biological activity [8].

Copigmentation, chemical and enzymatic acylation, and biosynthesis are effective strategies to stabilize anthocyanins [9]. In addition, the storage stability and the shelf-life of anthocyanin-related foods could be enhanced via complexing protein and anthocyanins [10]. The protein–polyphenol interactions were investigated between casein, β-lactoglobulin, SPP, black soybean protein isolate, and anthocyanins [1,10,11,12]. Both whey protein isolates [13] and egg ovalbumin (OVA) [14] could enhance the thermal stability of anthocyanins at a pH less than 7. However, the interaction of food components is complex and bidirectional, and the effects on proteins need to be considered to reveal the mechanism more comprehensively.

Lentils (Lens culinaris) contain around 20.6–31.4% protein, which consists of 16% albumins, 70% globulins, 11% glutelins, and 3% prolamins [15], while the globulin mainly constitutes egumin (11S) and vicilin (7S) proteins [16]. In some cases of doughnuts [17] and cake [18], lentil protein isolate (LPI) was added as a substitute for eggs/milk proteins because of its high digestibility. Meanwhile, LPI could be used for nano emulsion [19] and encapsulation [20]. Although LPI has been widely used in food processing, studies on the interaction phenomena between LPI and bioactive anthocyanins, especially C3G, are still lacking.

In this study, the physicochemical characteristics of LPI-C3G complexes were measured using dynamic light scattering and UV–vis absorption. Various spectroscopic analysis methods were used to investigate the interaction mechanism and the binding parameters including the inter-molecular forces of LPI to C3G and the secondary structure of LPI after adding C3G. Furthermore, the effects of LPI on thermal and oxidation stability of C3G and antioxidant activity of the LPI-C3G complexes were evaluated. These contributions are expected to reveal the interaction mechanism between anthocyanins and LPI, and provide the solution to stabilizing bioactive compounds in functional foods.

## 2. Materials and Methods

### 2.1. Materials and Reagents

C3G (98% purity) was obtained from Chengdu Lemeitian Pharmaceutical Technology Co., Ltd. (Chengdu, China). Lentils were obtained from the Trading Company of Lianyungang Luerjia (Lianyungang, China). Finally, 8-anilino-1-naphthalenesulfonic acid ammonium salt (ANS, 97%) was obtained from Sigma-Aldrich Co. (St Louis, MO, USA). All other chemicals used were of analytical grade.

### 2.2. Preparation of LPI

Whole lentil seeds were ground in a high-speed multifunctional crusher (KYS-0831, Keyingshang, Jinhua, China) to yield a lentil flour. The powder was stirred in n-hexane (1:3 *w/v*) for 60 minutes to remove the fat and the defatting process was repeated three times. The residue after defatting (three times) was placed in a fume hood for 48 h to evaporate the remaining n-hexane.

The LPI was prepared according to Aryee and Nickerson [21]. Briefly, the defatted lentil powers were dissolved in deionized water (1:10, *w/v*), and then the pH was adjusted to 9.0 with 1 M NaOH. After stirring for 2 h, the suspension was centrifuged at 4800 rpm for 20 min to collect the supernatant and residue was repeated twice. All supernatants were combined and the pH adjusted to 4.5 and left overnight at 4 ℃ to precipitate the protein. The protein was gathered by centrifugation, suspended in DI water at pH 7.0, and dialyzed in a dialysis bag with a molecular weight cut-off of 8000–10000 kDa against DI water for 72 h. Then, the dialysate was freeze-dried to obtain LPI and stored at −20 ℃.

### 2.3. Preparation of LPI-C3G Complexes

The LPI-C3G complexes were fabricated according to Nagy, Courtet-Compondu, Williamson, Rezzi, Kussmann, and Rytz [22] with slight modifications. Briefly, the LPI was dispersed in sodium phosphate buffer (10 mM, pH 6.3) and stirred using a magnetic agitator (HJ-6A, Guohua, Changzhou, China) with different concentrations of C3G for 2 h at 298 K away from light. The concentration of the protein solution was 0.5 mg/mL and the C3G concentrations were 0, 0.02, 0.04, 0.08, 0.16, and 0.20%. The group without C3G (0%) served as a control.

### 2.4. LPI-C3G Complex Particle Characteristics

The particle size and ζ-potential of the LPI-C3G complex were measured with Zetasizer Nano ZES (Malvern Instruments, Worcestershire, UK) at 298 K as described by Fu, Belwal, He, Xu, Li, and Luo [14]. Particle size was expressed by the z-average value.

### 2.5. Turbidity of the LPI-C3G Complexes

The turbidity of the LPI-C3G complexes was measured using a TU-1900 spectrophotometer (Persee, Beijing, China) [23]. Briefly, the LPI-C3G sample was dissolved in deionized water and the absorbance was recorded at 600 nm after placing at 20 ℃ for 24 h.

### 2.6. Foaming Performance of Complexes

The foaming properties were analyzed according to Shevkani, Singh, Kaur, and Rana [24] with slight modifications. Here, 10 mL LPI-C3G complexes were homogenized (15,000 rpm, 1 min). The foam volume at 0 min and 30 min was recorded. The foam capacity (FC) was calculated as the percentage of volume increase after suspension mixing; foam stability (FS) was estimated as the percentage of foam remaining after 30 min.

### 2.7. Emulsifying Activity and Emulsion Stability

The emulsifying activity index (EAI) and emulsion stability index (ESI) of the LPI-C3G complexes were measured using the method of Chen, Wang, Feng, Jiang, and Miao [25]. Briefly, 3 mL soybean oil was added to 9 mL of the prepared LPI-C3G complexes and dispersed at 10000 r/min for 1 min. Then, 50 μL of the emulsion was immediately transferred to a test tube containing 5 mL of 0.1% SDS (*w/v*) after homogenization. The absorbance of the samples was immediately measured at 500 nm after mixing at 0 and 10 min with the blank of the same concentration of SDS solution via a TU-1900 spectrophotometer (Persee, Beijing, China).

### 2.8. Sodium Dodecyl Sulfate-Polyacrylamide Gel Electrophoresis (SDS-PAGE)

SDS-PAGE was operated based on the method of Sui, Sun, Qi, Zhang, Li, and Jiang [26]. Briefly, the denatured LPI-C3G complex (10 ug) was added to the gel. The proteins were separated at different voltages (32 mV and 64 mV). After the completion of electrophoresis, the gel was stained with 0.10% Coomassie Brilliant Blue R-250 solution (methanol: acetic acid: water = 5:2:13 *v*/*v*/*v*) followed by de-staining with a mixture of acetic acid, ethanol, and water (*v*:*v*:*v* = 2:1:17) until the background was clear. The low MW protein marker (MW 14.4 to 97.4 kDa) from Beijing Solaibao Biotechnology (Beijing, China) was used as a molecular marker.

### 2.9. UV, Intrinsic Fluorescence, Synchronous Fluorescence, and Fourier-Transform Infrared (FTIR) Spectroscopy

The UV spectra were detected with a TU-1900 spectrophotometer under a wavelength range of 230–350 nm. Intrinsic fluorescence was determined with a Hitachi F-7000 fluorophotometer (Kyoto, Japan) according to a previously described methodology [27]. Intrinsic fluorescence spectroscopy of LPI with different concentrations of C3G was recorded at an excitation wavelength of 280 nm and an emission wavelength of 340–500 nm at 298 K, 308 K, and 318 K, respectively. The excitation and emission slit widths were 2.5 nm. 

Synchronous fluorescence spectra were detected according to a previously described methodology [28]. Briefly, the samples with and without C3G were recorded from 280–330 nm and 300–400 nm for the difference between the excitation and emission wavelength (∆λ) of 15 and 60 nm at 298 K, respectively.

The FTIR was detected with a Nicolet iS50 FTIR spectrometer (Thermo Fisher, Bremen, Germany) according to the previous study [29]. Briefly, mixture of 2.0 mg freeze-dried sample and 198 mg pure potassium bromide (KBr) powder was ground into a fine powder, pressed into pellets, and measured by FTIR. The spectra were performed in the region from 4000 to 400 cm^−1^ with a 4 cm^−1^ resolution and an accumulation of 64 scans. A pellet prepared with pure KBr powder was used as a baseline.

### 2.10. Circular Dichroism (CD) Spectroscopy

CD spectra were performed on an MOS-450 spectropolarimeter (Bio-Logic, Claix, France) in the far-UV region (190–250 nm) using a 1 mm cell at 298 K [10]. The sample was measured with a scan speed of 100 nm per minute and a constant nitrogen flush used through the scanning. The secondary structure was calculated using an online website: http://dichroweb.cryst.bbk.ac.uk (accessed on 17 August 2021) [30].

### 2.11. Surface Hydrophobicity

The surface hydrophobicity was determined with ANS and a fluorescence spectrophotometer (F-7000, Hitachi, Kyoto, Japan), and the specific operation process was as previously reported by Dai, Chen, Li, Li, Hu, Liu, and Li [31]. The excitation and emission slit were 5.0 nm. The excitation wavelength was 390 nm and the emission spectra were from 400 nm to 600 nm. Next, 20 μL 8 mmol/L ANS solution was added into a 4.0 mL sample and kept in the dark at room temperature for 10 min. C3G solution was used as a blank to correct the background of the fluorescence. The surface hydrophobicity was remarked as the relative ANS-fluorescence intensity.

### 2.12. Differential Scanning Calorimeter (DSC) 

DSC was performed as reported by Parolia, Maley, Sammynaiken, Green, Nickerson, and Ghosh [32] with slight modifications. A 3 mg sample was weighed in an aluminum crucible and sealed. The crucibles were heated from 25 to 120 ℃ at a constant rate of 5 ℃ min^−1^ and a constant purge of dry nitrogen gas at 20 mL min^−1^. The peak temperature of denaturation was acquired from the thermal curve using TA 60 software.

### 2.13. Fluorescence Quenching Mechanism, Binding Constant, and Thermodynamic Parameters

Collisional quenching of fluorescence was described by the Stern–Volmer equation [33]:

F_0_/F = 1+K_q_τ_0_[Q] = 1+K_sv_[Q] (1)

where F_0_ and F were the fluorescence intensities with and without quencher, respectively; τ_0_ was the fluorescence lifetime of the fluorophore in the absence of quencher and equaled 10^−8^ s; K_q_ was the bimolecular quenching rate constant; and [Q] was the molar concentration of quencher and K_sv_ was the Stern–Volmer quenching constant, which could be given by K_sv_= K_q_τ_0_. Therefore, K_sv_ could be obtained by the linear regression of a plot of F_0_/F against [Q].

The double logarithmic Stern–Volmer equation (Equation (2)) was used to calculated the binding constant (Ka) and binding site numbers (n) [34].

Log(F_0_ − F)/F = LogKa + nLog[Q] (2)

The thermodynamic parameters were calculated with the Van ’t Hoff Equations (3)–(4) [35]:

lnKa = −∆H/RT + ∆S/R(3)

∆G = ∆H − T∆S(4)

where ∆H is the enthalpy change; ∆S is the entropy change and ∆G is the free energy change; R is the gas constant (8.314 J/moL·K); T is the absolute temperature (K); and ∆H and ∆S could be determined from the slope and intercept of the linear regression curve of lnKa versus (1/T).

### 2.14. Thermal and Oxidation Stability 

The thermal and oxidation stability were determined by referring to the method of He, Xu, Zeng, Qin, and Chen [36]. Briefly, the C3G content was determined at 537 nm with UV–vis spectrophotometry after the sample was heated in the water bath (80 ℃) or oxidized with hydrogen peroxide (0.05 mg/mL) for 2 h. 

### 2.15. Antioxidative Properties of the LPI-C3G Complexes

The antioxidant activity of the LPI-C3G complexes was determined by a ferric-reducing antioxidant potential (FRAP) kit (Biyuntian, Shanghai, China).

### 2.16. Statistical Analysis

All experiments were carried out in triplicate and the data were expressed as mean ± standard deviation. One-way analysis of variance (ANOVA) of the data was performed using GraphPad Prism 5.0. The level of *p* < 0.05 was considered statistically significant.

## 3. Results and Discussion

### 3.1. Physical Properties of LPI-C3G Complexes

**Figure 1:**
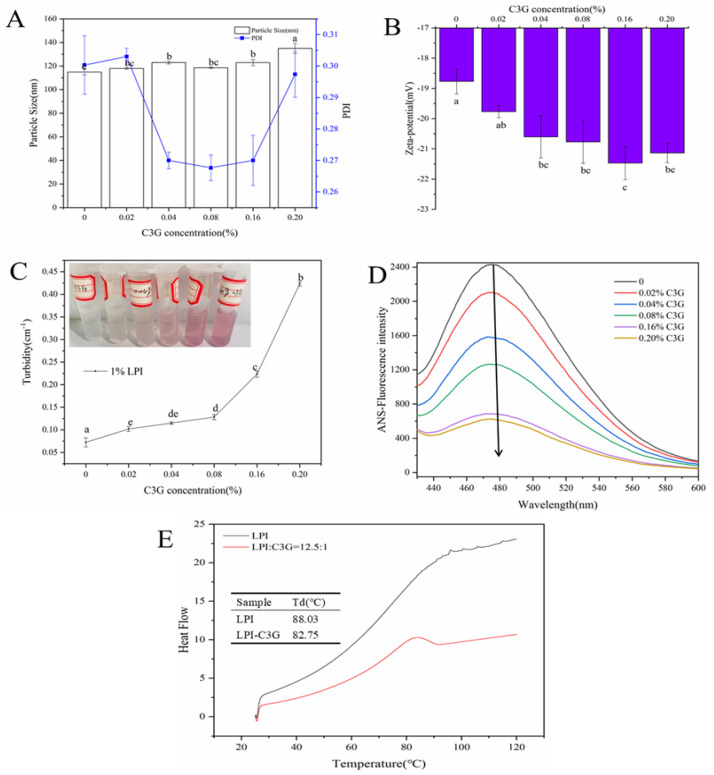
Particle size and PDI (**A**), zeta potential (**B**), and turbidity (**C**) of LPI-C3G complexes at different C3G concentrations; ANS-fluorescence intensity (**D**) and thermal denaturation temperatures (**E**) of LPI-C3G complexes (0.5 mg/mL LPI). Different letters represent significant differences (*p* < 0.05).

*Particle size*. The polymer polydispersity index (PDI) values of the LPI-C3G complexes were less than or equal to 0.3 except the second group (0.02% C3G) (Figure 1A), indicating that the particle size was uniformly distributed [37]. The average particle size of the LPI-C3G complexes increased significantly (*p* < 0.05) from 115 ± 2.31 to 135 ± 4.36 nm. When LPI interacts with C3G, the hydrophobic groups of the protein are exposed, which facilitates the binding of C3G to the protein, forming small soluble polydisperse particles, and with increasing C3G concentration, these particles may aggregate into larger particles [13,38]. Similarly, the average size in acid and neutral pH increased significantly after adding C3G in egg ovalbumin due to the aggregation of egg ovalbumin [14].

*Zeta potential*. Zeta potential has a critical role in the stability of LPI-C3G complex dispersion. As shown in Figure 1B, it could be seen that the LPI-C3G complexes have negative charges, which indicates that there might be electrostatic repulsion between the LPI-C3G complexes. Meanwhile, the absolute value of all LPI-C3G complexes except the sixth group (0.2% C3G) increased with the increase in the C3G content, which was consistent with the reported study [26]. The binding of negatively charged C3G with the protein results in an increase in the net charge on the surface of the complex to resist aggregation [39].

*Turbidity*. The turbidity of LPI with or without C3G is shown in Figure 1C. The turbidity was at its minimum in the control group (0% C3G); by contrast, the turbidity increased significantly (*p* < 0.05) after different concentrations of C3G were added, which might be ascribed to the microaggregation between C3G and LPI. Turbidity could be considered a complicated process affected by particle size, color, refractive index, and particle interactions including micro- and nano-aggregation [40]. Meanwhile, no obvious precipitation or sedimentation could be seen after 24 h at room temperature (Figure 1C inset), manifesting the good stability of LPI-C3G complexes and resistance to gravitational separation.

*Surface hydrophobicity*. The surface hydrophobicity was determined with ANS and the results are summarized in Figure 1D. The surface hydrophobicity of LPI decreased with the increase of C3G concentration. The remarkable decrease was considered to be related to the C3G binding with LPI, leading to the binding sites of ANS being reduced and an increase in the surface polarity [41]. In addition, the hydrophilic groups introduced by C3G might increase the protein’s surface hydrophilicity [14].

*Thermal stability*. The thermal stability of the LPI-C3G complexes was detected with DSC. The Td value of LPI was 88.03 °C. After interacting with C3G, the Td value of LPI shifted to 82.75 °C (Figure 1E). Td is an indicator of protein thermal stability, strongly dependent on its spatial structure [42]. The lower Td value indicated that the LPI structure might be changed, caused by C3G. C3G enhanced the thermal stability of soybean protein (7S and 11S) at pH 7.0 [43].

### 3.2. Functional Properties of LPI-C3G Complexes

**Figure 2:**
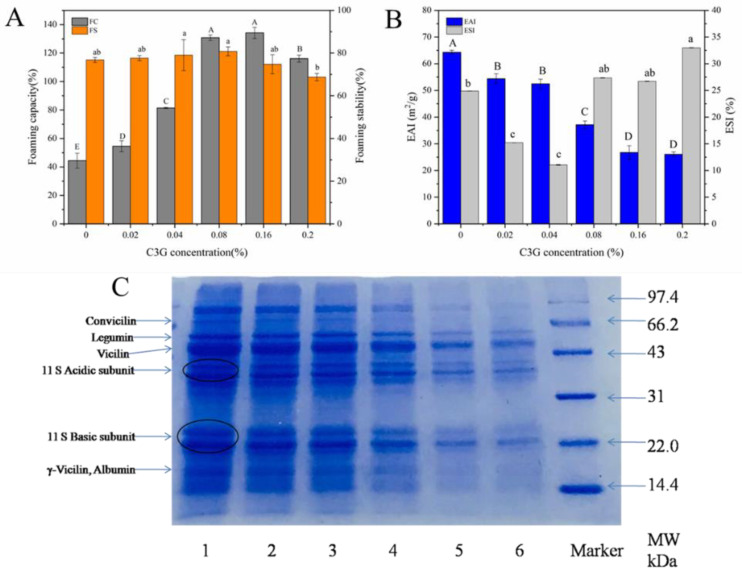
Foaming capacity and stability (**A**), emulsifying activity index (EAI) and emulsion stability index (ESI) (**B**), and SDS-PAGE profiles (**C**) of LPI-C3G complexes at different C3G concentrations. Lanes 1, 2, 3, 4, 5, and 6 represent C3G concentrations of 0, 0.02%, 0.04%, 0.08%, 0.16%, and 0.2%, respectively. Different letters represent significant differences (*p* < 0.05).

*Foaming properties*. The functional properties of proteins are affected by their spatial conformation and surface charge [26]. The FC of LPI-C3G complexes were significantly (*p* < 0.05) improved compared to LPI (Figure 2). It might be that the interaction of LPI and C3G changed the interfacial properties of the protein film, forming an elastic and stable interfacial film on the air/water surface. Anthocyanins could enhance the foaming properties of soybean protein isolates [26]. Meanwhile, it has also been reported that the binding between polyphenols and proteins, such as tannins and sodium caseinate, actually reduces the foamability of proteins [44]. The FS of LPI-C3G complexes did not change significantly compared to LPI, although there was a slight decrease at high C3G concentrations (0.16% and 0.2%) (Figure 2A). FC and FS are complex phenomena that depend on the adsorption capacity of proteins at the air/water interface, interfacial rheological properties, and the diffusion rate of the gas in the foam [44].

*Emulsifying properties.* The emulsifying ability index (EAI) of the LPI-C3G complexes was significantly (*p* < 0.05) decreased compared with the LPI group. The emulsifying stability index (ESI) first decreased and then increased, which might be related to the effect of C3G on the LPI structure (Figure 2B). These results suggested that the addition of C3G might reduce the emulsifying capacity of LPI, but improve its ability to maintain the emulsion structure [45]. The changes in these functional properties affect their application in food processing.

### 3.3. Effect of C3G on the Molecular Weight and Secondary Structure of LPI

*SDS-PAGE analysis.* The SDS-PAGE results of LPI and LPI-C3G are given in Figure 2C. All sample bands exhibited molecular weights ranging from roughly 14.4 to 97 kDa. The bands at ~70 kDa, ~60 kDa, and ~50 kDa could be attributed to convicilin legumin subunits and vicilin subunits, respectively. Separate bands at ~40 kDa and ~20 kDa were acidic and basic subunits of legumin, respectively [46]. Moreover, bands between 22 and 14.4 kDa were identified as γ-vicilin and various albumin polypeptides [47]. Compared to LPI, the bands of 11S basic subunit and acid subunit, vicilin, and legumin appeared in LPI-C3G complexes, which suggested that C3G might react with polypeptides of LPI. Studies have shown that polyphenol rings could have hydrophobic interactions with the hydrophobic cavities of LPI [48]. Meanwhile, a high concentration of C3G caused the protein bands to weaken or even disappear, and no new bands appeared, which indicated that no oligomer was formed in the LPI-C3G complexes and the quaternary structure of LPI was complete [26].

**Figure 3:**
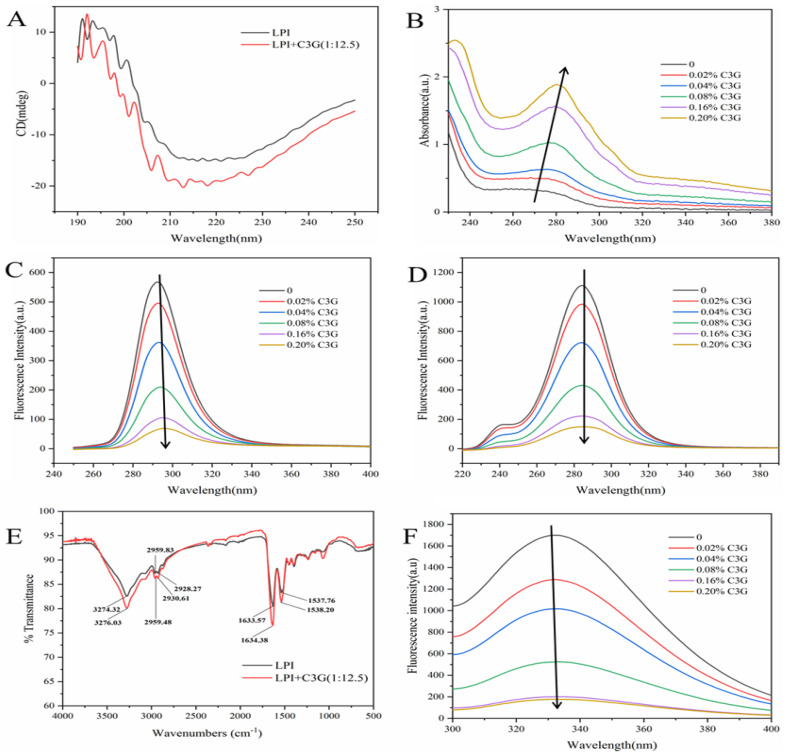
CD spectra (**A**), UV–vis spectra (**B**), synchronous fluorescence spectra at ∆λ = 15 nm (**C**) and ∆λ = 60 nm (**D**), FTIR spectra (**E**), and intrinsic fluorescence spectra (**F**) of LPI-C3G complexes (0.5 mg/mL LPI) at 298 K.

*CD spectra of the LPI-C3G complexes*. The far-ultraviolet CD spectra of LPI and LPI-C3G complexes are presented in Figure 3A. The CD spectra of LPI exhibited a negative peak at 204–218 nm, indicating that the β-sheet was predominant in LPI conformational after binding with C3G [28]. The free LPI contained about 9.13% α-helix, 22.77% β-turn, 38.77% β-sheet, and 29.80% random coil (Table 1). The content of α-helix in the LPI-C3G complex reduced to 7.80%, which might be owing to C3G inserting itself into the hydrophobic surfaces of LPI molecules destructing hydrogen bonding networks [49]. It was in accordance with the result of surface hydrophobicity (Figure 1D). The random coil content increased to 31.50% after adding C3G, which might be because the protein peptide chain was loosened and stretched after interacting with C3G [39].

### 3.4. UV–Vis Spectra of LPI-C3G Complexes

UV–vis spectra are often used to investigate structural changes and intermolecular interactions [49]. When the concentration of C3G increased from 0% to 0.2%, the absorbance of LPI enhanced and the maximum absorption peak shifted from 268 nm to 280 nm (Figure 3B), which might be the interaction between the aromatic residues of LPI and C3G contributing to the transition of the more hydrophobic microenvironment of the aromatic amino acid residues in proteins [29]. Similar results were reported between black soybean protein isolate with C3G, which showed a very small redshift from 272 nm to 279 nm [11].

### 3.5. Synchronous Fluorescence Spectra of LPI-C3G Complexes

The microenvironment near the chromophore molecule and the conformational information of the molecule was detected with synchronous fluorescence. As shown in Figure 3C,D, tyrosine (Tyr) and tryptophan (Trp) residues exhibited characteristic signals at 15 and 60 nm, respectively [14]. When the concentration of C3G increased, the synchronous fluorescence intensity of LPI decreased progressively, followed by a redshift (292 to 296 with ∆λ at 15 nm and 284 to 285 nm with ∆λ at 60 nm), which indicated that the hydrophobicity around the Tyr and Trp residues of LPI decreased, and it might be that the Tyr and Trp residues were exposed to an aqueous or hydrophilic environment after binding with C3G [27]. Compared with Tyr, Trp showed stronger fluorescence intensity, which might be because it was closer to the binding site in the process of interaction. In short, the microenvironmental changes of Tyr and Trp revealed the changes of their conformation after binding with C3G.

### 3.6. FTIR Spectra of LPI-C3G Complexes

As shown in Figure 3E, at 3274.32 cm^−1^ of LPI, it could be associated with the O-H stretching vibration of hydroxyl-bound water [50]. However, a shift occurred after complexation with C3G, and the peak was at 3276.03 cm^−1^, which might be that the hydrogen bonds were formed between the amide group of aspartates in LPI and the hydroxyl groups in C3G [51]. In addition, the FTIR spectra for LPI showed absorption at 2959.83 cm^−1^ and 2928.27 cm^−1^, which might be the C-H stretching vibrations. With the addition of C3G, its absorption peaks shifted to 2959.48 cm^−1^ and 2930.61 cm^−1^. The absorption bands at 1600–1700 cm^−1^ were amide I resulting from the stretching vibration of the C-O and C-N groups; meanwhile, amide II occurred in the region <1548 cm^−1^, which was mainly dominated by the bending vibration of the N-H groups and the stretching vibrations of the C-N groups [29,51]. The amide I shifted from 1633.57 to 1634.38 cm^−1^, and that of amide II shifted from 1537.76 to 1538.20 cm^−1^ after C3G added (Figure 3E), which implied that the secondary structure of LPI was changed owing to the interaction with C3G. In addition, the intensity reduction might be related to a decrease of α helix, electrostatic repulsion, and hydrophobic action [27,28,51].

### 3.7. Intrinsic Fluorescence Analysis

Figure 3F showed the fluorescence spectra of LPI-C3G complexes at 280 nm. The λmax value of LPI was around 332 nm. When the C3G concentration increased, the fluorescence intensity of LPI decreased progressively with a redshift from 332 to 334 nm, which might be related to the increased polarity of the microenvironment around Trp and Tyr residues in LPI caused by the reaction of C3G and LPI [12,29]. C3G could concentration-dependently quench the intrinsic fluorescence of LPI [38]. Consistent results were observed in the study of C3G with egg ovalbumin [14], as well as silkworm pupae protein [12].

### 3.8. Fluorescence-Quenching Mechanism and Binding Constant 

Generally, the fluorescence-quenching mechanism includes dynamic and static [33]. With the increase in temperature, the dynamic quenching constant increases, while the static quenching constant decreases, which can be used to distinguish the quenching types [1,52].

**Figure 4:**
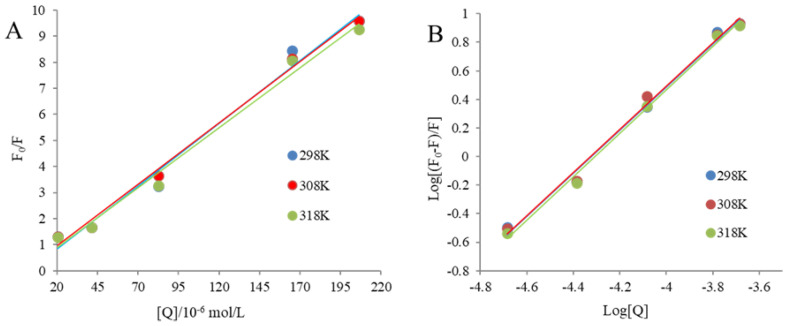
Stern–Volmer plots of LPI quenched by C3G (0–0.2%) at 298 K, 308K, and 318K (**A**), and the double-logarithmic regression plots of 0.5 mg/mL LPI in the presence of C3G (0–0.2%) at 298 K, 308K, and 318K (**B**).

A Stern–Volmer plot of LPI quenched by C3G at 298, 308, and 318 K was acquired based on Equation (1) (Figure 4A and Table 2). With the increase in temperature, the Ksv values of LPI reduced, and the Kq values were significantly (*p* < 0.05) higher than the limiting diffusion collision quenching constant value (2 × 10^10^ M^−1^ s^−1^), manifesting that the quenching mechanism for LPI by C3G was dominated by the static quenching [29]. This result was mutually verified with UV absorption spectroscopy. In dynamic quenching, the absorption spectra of fluorescent substances do not change, while static quenching is the opposite [53].

The binding constant (Ka) and binding site numbers (n) are summarized in Figure 4B and Table 2, respectively. Markedly, the curves of binding affinity presented good linear relationships (R^2^ > 0.99), and the K_a_ for LPI and C3G was on the order of 10^6^, which was much higher than that reported for the interaction between C3G and β-Lactoglobulin, as well as egg ovalbumin binding with C3G (10^3^–10^4^ M^−1^) [10,14]. The Ka value increased with the rising temperature, indicating that LPI has a strong binding affinity for C3G, and this was an endothermic reaction (Table 2) [28]. There might be 1.5 binding sites of LPI involved in the binding process.

### 3.9. Thermodynamic Parameters and Binding Forces between LPI and C3G 

As shown in Table 2, all ∆G was negative, which means that LPI and C3G spontaneously interacted with each other. In addition, the ∆H and ∆S values for the binding of C3G and LPI were 4.61 and 140.01 J·mol^−1^, respectively, implying that the interaction was dominated by hydrophobic effects and the process was endothermic [54]. Similar results were reported about β-Lactoglobulin with C3G [10], as well as malvidin-3-O-glucoside [28].

### 3.10. Stability of C3G Influenced by LPI 

The influence of LPI on the thermal and oxidation stability of C3G is summarized in Table 3. It was found that there was a significant decrease in C3G content, but the LPI-C3G complexes were remarkably higher compared with C3G alone (*p* < 0.05), and the remaining C3G content had the highest LPI concentration (0.2 mg/mL). When the LPI concentration increased, the degradation rate of C3G induced by thermal treatment and oxidation decreased from 46.29% to 4.52% and 71.13% to 32.58%, respectively, indicating that LPI could protect C3G from degradation. Soy protein isolate [29] and egg ovalbumin [14] were also found to have protective effects on C3G. Interestingly, heat treatment had a better protective effect than oxidation treatment, which might be because LPI dissociating at a high temperature was conducive to binding with anthocyanin [55]. Heating the protein might effectively protect against anthocyanin degradation. Studies have shown that heated silkworm pupae protein [12], SPI [29], and milk proteins (whey proteins and casein) [36] could better enhance the thermal and oxidation stability of anthocyanins.

Anthocyanin is unstable and sensitive to heat, light, pH, oxygen, and other environmental factors and the degradation of anthocyanins affects the sensory quality and reduces bioactivity [9]. Various studies have provided strong evidence that the interaction between proteins and anthocyanins could improve the stability of anthocyanins [1,12,28,29,36]. In this study, LPI and C3G might form a complex through hydrophobic interaction, which could effectively improve their thermal and oxidation stability.

### 3.11. Antioxidative Properties of LPI-C3G Complexes 

The antioxidant activity of LPI-C3G complexes was detected using the FRAP method. As shown in Table 3, regardless of heat or oxidation treatment, the reducing ability of C3G was higher than LPI-C3G complexes, and the higher the concentration of LPI, the weaker the reducing ability of the LPI-C3G complexes. These results suggested that conjugating C3G with LPI protects C3G from degradation, but inhibits its antioxidant activity. This might be because when polyphenols interact with proteins through hydrophobic forces, there are fewer opportunities for pro-oxidants in the environment to interact with polyphenol molecules [49].

## 4. Conclusions

The spectroscopic analyses indicated that LPI interacted with C3G and formed complexes via hydrophobic effects with Ka of 3.24 × 106 M^−1^ at 298 K and quenched the intrinsic of LPI by static quenching. The complexation changed the particle size and surface charge of proteins in LPI, but considerably decreased their surface hydrophobicity, as well as affecting the functional properties (foaming and emulsifying properties). Meanwhile, LPI enhanced the thermal and oxidative stability of C3G, but inhibited its antioxidant activity. These effects might be related to changes in their structure during the interaction. Overall, this study highlights the LPI in improving the stability of anthocyanins, and LPI–nutrient complexes might help stabilize bioactive compounds and develop functional foods.

## Figures and Tables

**Table 1:** CD analysis of the secondary structure of LPI with and without C3G at 298 K.

Sample	α-Helix (%)	β-Sheet (%)	β-Turn (%)	Random Coil (%)
LPI	9.13 ± 0.15	38.77 ± 0.23	22.77 ± 0.50	29.80 ± 0.85
LPI-C3G	7.80 ± 0.17	38.47 ± 0.35	22.57 ± 0.25	31.50 ± 0.20

**Table 2:** Quenching constant, binding constants, and thermodynamic parameters of LPI-C3G complexes at 298, 308, and 318 K.

Complex	T (K)	K_sv_ (×10^4^ M^−1^)	K_q_ (×10^12^ M^−1^·s^−1^)	R ^2a^	K_a_ (×10^6^ M^−1^)	*n*	R ^2b^	ΔH (kJ·mol^−1^)	ΔG (kJ·mol^−1^)	ΔS (J·mol^−1^)
LPI-C3G	298	4.83 ± 0.01 a	4.83 ± 0.01 a	0.9828	3.24 ± 0.01 a	1.506	0.9916		−37.11	
	308	4.76 ± 0.03 b	4.76 ± 0.03 b	0.9929	3.33 ± 0.04 b	1.508	0.9913	4.61	−38.51	140.01
	318	4.62 ± 0.02 c	4.62 ± 0.02 c	0.9867	3.63 ± 0.02 c	1.532	0.9942		−39.91	

R ^2a^ is the correlation coefficient for the K_sv_ value; R ^2b^ is the correlation coefficient for the K_a_ value. Different letters (a, b and c) in the same column indicate significant differences (*p* < 0.05).

**Table 3:** Effects of LPI on stability and antioxidant activity of LPI-C3G complexes.

Treatment	LPI Concentration (mg/mL)	C3G (mg/L)	C3G Degradation Rate (%)	FRAP Assay (mM FeSO_4_/g DW)
Untreated	0.00	41.33 ± 0.12		
Heated at 80 ℃ for 2 h	0.00	22.20 ± 0.35 e	46.29 ± 0.01 a	1.58 ± 0.04 a
	0.05	28.07 ± 0.23 c	32.10 ± 0.01 c	1.45 ± 0.06 ab
	0.10	25.40 ± 0.20 d	38.55 ± 0.00 b	1.42 ± 0.07 ab
	0.15	38.13 ± 0.31 b	7.74 ± 0.01 d	1.27 ± 0.30 ab
	0.20	39.47 ± 0.23 a	4.52 ± 0.01 e	1.19 ± 0.01 b
Oxidized by 0.05 mg/mL H_2_O_2_ for 2 h	0.00	11.93 ± 0.50 e	71.13 ± 0.01 a	1.21 ± 0.03 a
	0.05	15.93 ± 0.42 c	61.45 ± 0.01 c	1.18 ± 0.01 a
	0.10	14.73 ± 0.12 d	64.35 ± 0.00 b	1.14 ± 0.06 ab
	0.15	26.73 ± 0.31 b	35.32 ± 0.01 d	1.06 ± 0.01 ab
	0.20	27.86 ± 0.12 a	32.58 ± 0.00 e	1.00 ± 0.13 b

Values are expressed as the mean ± standard deviation. Different letters in the same column indicate significant differences (*p* < 0.05).

## Data Availability

Data is contained within the article.
